# JAK-STAT in Early Hematopoiesis and Leukemia

**DOI:** 10.3389/fcell.2021.669363

**Published:** 2021-05-14

**Authors:** Eirini Sofia Fasouli, Eleni Katsantoni

**Affiliations:** Basic Research Center, Biomedical Research Foundation, Academy of Athens, Athens, Greece

**Keywords:** JAK-STAT, STATs, hematopoiesis, hematopoietic stem cells, leukemia, STAT5

## Abstract

Hematopoietic stem cells (HSCs) produce all the terminally differentiated blood cells and are controlled by extracellular signals from the microenvironment, the bone marrow (BM) niche, as well as intrinsic cell signals. Intrinsic signals include the tightly controlled action of signaling pathways, as the Janus kinase-signal transducer and activator of transcription (JAK-STAT) pathway. Activation of JAK-STAT leads to phosphorylation of members of the STAT family to regulate proliferation, survival, and self-renewal of HSCs. Mutations in components of the JAK-STAT pathway are linked with defects in HSCs and hematologic malignancies. Accumulating mutations in HSCs and aging contribute to leukemia transformation. Here an overview of hematopoiesis, and the role of the JAK-STAT pathway in HSCs and in the promotion of leukemic transformation is presented. Therapeutic targeting of JAK-STAT and clinical implications of the existing research findings are also discussed.

## Introduction

**FIGURE 1:**
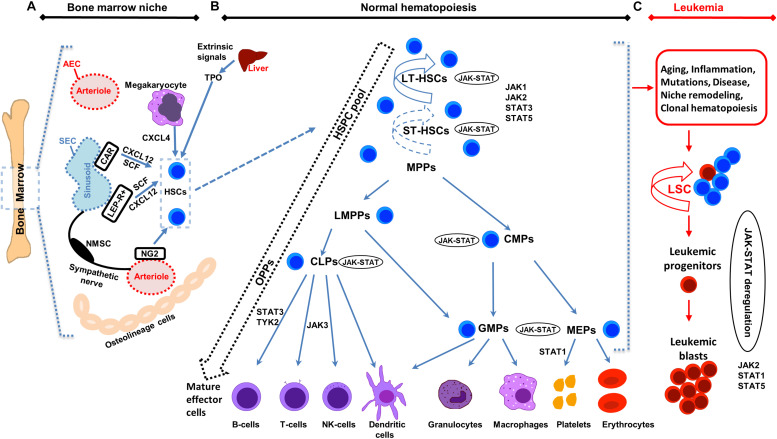
Illustration of bone marrow niche, normal and leukemic hematopoiesis. **(A)** Schematic representation of the bone marrow (BM) niche summarizing key cell types and functional features. HSCs reside in the proximity of BM vessels (arterioles or sinusoids). Mesenchymal stem cell (MSC) populations include, among others, NG2 +, LEPR + and CAR cells and promote HSCs maintenance by releasing important factors (i.e., CXCL12, SCF). Endothelial cells (ECs) (arteriolar endothelial cells (AECs), surrounding the arterioles and sinusoidal endothelial cells (SECs), surrounding the sinusoids) also release important factors for HCSs maintenance. Sympathetic nerve fibers regulate HSCs migration through the sinusoids. MSC subpopulations, ECs, non-myelinating Schwann cells (NMSCs) and HSC progeny (i.e., megakaryocytes) contribute to the regulation of HSC homeostasis or regenerative hematopoiesis. Megakaryocytes produce CXCL4 to regulate HSCs. **(B)** Schematic representation of normal hematopoiesis. HSCs reside at the top of the hierarchy. Differentiation is considered today more of a continuum, than a step-by-step procedure, represented by the dashed arrow on the left. The HSC pool is heterogeneous in terms of self-renewal and differentiation properties. Self-renewal of HSCs is denoted by an arrow around the cells (solid arrow represents strong and dashed arrow weaker self-renewal potential). Hematopoietic Stem and Progenitor cells (HSPCs) pool contains long-term self-renewing HSCs (LT-HSCs), short-term self-renewing HSCs (ST-HSCs) and non-self-renewing multipotent progenitors (MPPs). Throughout differentiation, HSCs might first lose self-renewal capacity and then lineage potential as they commit to evolving to a mature functional cell of a specific lineage. MPPs, might have unilineage, bi- or trilineage potential. MPPs advance to oligopotent progenitors (OPPs), including the lymphoid-primed multipotent progenitors (LMPPs), the common lymphoid progenitors (CLPs) and the common myeloid progenitors (CMPs). The myeloid and lymphoid compartments remain associated in the hierarchy *via* the lymphoid-primed multipotent progenitors (LMPP). CMPs give rise to megakaryocyte/erythrocyte progenitors (MEPs) and granulocyte/macrophage progenitors (GMPs). LMPPs give rise to give GMPs and CLPs. The OPPs through the lineage-restricted progenitors give rise to the mature effector cells (B-cells, T-cells and NK-cells, dendritic cells, granulocytes, macrophages, platelets, and erythrocytes). **(C)** Schematic representation of leukemic hematopoiesis. Aging, mutations, disease, inflammation, niche dysfunction/alterations and clonal hematopoiesis can lead to the generation of a leukemic stem cell (LSC). LSC can differentiate into the hematopoietic lineage carrying the mutation/s or remain as immature progenitor cells, called blast cells. Not all intermediate cell stages are depicted and cells are not in scale. Main differentiation points where the JAK-STAT pathway, JAKs and STATs exert their roles are shown.

Hematopoietic stem cells (HSCs) produce all the terminally differentiated blood cells (Figure 1) and are controlled by extracellular signals from the microenvironment or niche, and intrinsic cell signals that include signaling pathways. HSCs are ideal for advanced therapies, because of their multipotent and self-renewing properties. T he niche supports HSC maintenance, regulation, self-renewal and proliferation (Crane et al., 2017). Janus kinase-signal transducer and activator of transcription (JAK-STAT) pathway activation leads to phosphorylation of STATs that regulate hematopoiesis, and HSCs proliferation, survival and self-renewal. Dysregulation of the JAK-STAT pathway has been associated with various malignancies. STAT5, a member of the STAT family, controls normal lympho-myeloid development (Wang and Bunting, 2013) and plays a critical role in leukemia. Leukemia, characterized by overproduction of abnormal blood cells and defects in HSCs, is considered an age-related disease and its incidents rose continuously in the last decades (Hao et al., 2019). Albeit extensive research in this field, a lot of questions on the underlying molecular mechanisms of JAK-STAT in HSCs in normal lympho-myeloid development and leukemia remain unanswered. A better understanding of the mechanisms and signaling pathways in HSCs will contribute to improving already existing therapeutic approaches and design novel ones for hematopoietic malignancies. Here, a short overview of the advances on HSCs biology and the role of the JAK-STAT pathway in early hematopoiesis and leukemia, together with therapeutic implications of the existing research findings are discussed.

## Hematopoiesis

### Hematopoietic Stem and Progenitor Cells, and Aging

Hematopoiesis generates all the terminally differentiated cellular blood components (Figure 1). HSCs can be found reposed or they proliferate and differentiate, depending on their internal programming and the external signals from the microenvironment (Nakamura-Ishizu et al., 2014). HSCs have the unique potential for multi-potency and self-renewal (Seita and Weissman, 2010) and in adults are mainly situated in the bone marrow (BM). HSCs continuously replenish the blood throughout the lifetime (Orkin and Zon, 2008; Dzierzak and Philipsen, 2013) and can functionally reconstitute the entire blood system in an irradiated recipient by stem cell transplantation (Appelbaum, 2007). Hematopoietic stem and progenitor cells (HSPCs) pool contains long-term self-renewing HSCs (LT-HSCs), short-term self-renewing HSCs (ST-HSCs), and non-self-renewing multipotent progenitors (MPPs) (Seita and Weissman, 2010; Zhang et al., 2019). Distinct myeloid-biased MPP subsets work together with lymphoid-primed MPP cells to guide the generation of blood components. MPPs are produced in parallel by HSCs, at different levels and kinetics depending on the hematopoietic needs in normal or regenerating conditions. In the latter case, the myeloid-biased MPPs are overproduced by HSCs for a short time, to support myeloid amplification and rebuilding of the hematopoietic system (Pietras et al., 2015). The multi-lineage priming of MPPs is linked to low-level activation of expression programs for various lineages. Lineage fate choice is then connected with activation of a specific expression program while the rest are switched-off. Recent single-cell technologies have questioned the rigid past model of hematopoiesis of MPPs advancing to oligopotent progenitors (OPPs), and then to lineage-committed and mature effector cells. The fluidity of HSC differentiation is today represented more by a continuum than a rigid step-by-step procedure. Heterogeneous populations are organized hierarchically, with gradual highly flexible progression during differentiation (Figure 1; Laurenti and Gottgens, 2018; Jacobsen and Nerlov, 2019).

Mutations during HSCs development lead to leukemia, myelodysplasia, or BM failure. HSCs are susceptible to age-related changes triggered by intrinsic and extrinsic factors. Aged HSCs feature defective repopulating and homing capacity, increased mobilization and myeloid lineage-biased skewing, decreased fitness, and epigenetic/genetic changes (Lee J. et al., 2019). Many hematological malignancies, including acute myeloid leukemia (AML), are age-dependent. Aging is also connected to expanded clonal hematopoiesis (Konieczny and Arranz, 2018). High fitness of the young HSC pool serves to maintain the existing condition, while in an aged HSC pool the low fitness allows accumulation of mutations and epigenetic changes to improve fitness. For example, *Bcr-Abl* provides an advantage to aged B-lymphoid progenitors compared to young ones, leading to increased *Bcr-Abl* leukemogenesis (Henry et al., 2010, 2011). HSCs or other progenitors when undergoing a mutation can give rise to a leukemic stem cell (LSC), which features a dysregulated self-renewal program (Figure 1). LSCs differentiate into the hematopoietic lineage carrying the mutation/s or remain as immature progenitor cells, called leukemic blast cells (Buss and Ho, 2011; Hanekamp et al., 2017; Vetrie et al., 2020).

### Bone Marrow Niche

The BM niche constitutes a specialized microenvironment, composed of diverse cell types to support maintenance, induction, differentiation and proliferation of HSCs in embryos and adults. Definitive HSCs develop from the hemogenic endothelium within the aorta-gonad-mesonephros (AGM) region, then migrate to the fetal liver and finally to the adult BM (Gao et al., 2018). Single-cell transcriptomics analysis has defined two molecularly distinct arterial endothelial cell (AEC) populations and putative HSC-primed hemogenic endothelial cells (HECs) in the dorsal aorta of the AGM region, whose number peaked at mouse embryonic day (E) 10.0 and decreased thereafter (Hou et al., 2020). Primitive vascular endothelial cells (ECs) from E8.0 experienced an initial arterial fate choice to become HSC-primed HECs, followed by a hemogenic fate conversion (Hou et al., 2020). Similarities in the development of HSC-primed HECs between mouse and human embryos exist (Zeng et al., 2019; Hou et al., 2020).

The BM niche includes mesenchymal stem cells (MSCs), ECs [AECs and sinusoidal ECs (SECs)], osteolineage cells (OLCs), non-myelinating Schwann cells and progeny of HSCs (such as megakaryocytes and macrophages) located together with the HSCs in the extracellular matrix (Figure 1; Yu and Scadden, 2016; Mendez-Ferrer et al., 2020; Mitroulis et al., 2020). Different niche cell populations regulate the balance between HSC proliferation and quiescence during homeostasis or regenerative hematopoiesis. Identification of MSCs, which are important for HSCs function, has relied on reporter mouse models for markers including Nestin (NES), Stem Cell Factor (SCF), CXC chemokine ligand 12 (CXCL12), nerve/glial antigen 2 (NG2), and Leptin receptor (LEPR) (Sugiyama et al., 2006; Ding et al., 2012; Sugiyama and Nagasawa, 2012; Ding and Morrison, 2013; Kunisaki et al., 2013). NG2^+^ pericytes, found spatially linked to arteriolar niches, have been confirmed to be important for the maintenance of HSC quiescence (Kunisaki et al., 2013). HSCs are localized predominantly in the perisinusoidal space and in close proximity to Leptin Receptor^+^Cxcl12^high^ cells (Acar et al., 2015). Adipo-osteogenic progenitors have been also found essential for HSCs proliferation and maintenance in an undifferentiated state (Omatsu et al., 2010). Depletion of CXCL12-abundant reticular (CAR) cells *in vivo* has led to severe impairment of the adipogenic/osteogenic differentiation competency, and reduced SCF and CXCL12 production, resulting in decreased lymphoid and erythroid progenitors cycling (Omatsu et al., 2010). ECs and *Lepr*-expressing perivascular cells, through the expression of essential factors, such as SCF, maintain HSCs and *Scf* deletion from both endothelial and *Lepr*-expressing cells has led to HSCs depletion from the BM (Ding et al., 2012). *Foxc1* has been found significant for the development and maintenance of the mesenchymal niches, through enhancement of CAR cell development by upregulation of CXCL12 and SCF expression (Omatsu et al., 2014). Megakaryocytes have been also found to control HSCs quiescence by producing CXCL4 (Bruns et al., 2014). Single-cell RNA-seq has characterized in detail the mouse BM stroma in homeostasis and leukemia. Seventeen distinct cell populations have been defined, including MSCs, OLCs, chrondrocytes, fibroblasts, pericytes, and EC subsets, together with new differentiation paths (Baryawno et al., 2019). The dynamic and diverse transcriptional landscape of vascular, perivascular, and osteoblast BM niche cell populations has been confirmed both at homeostasis and stress hematopoiesis (Tikhonova et al., 2019). Vascular-endothelial Delta-like Notch ligand 4 (*Dll4*) expression regulates HSC differentiation and lineage commitment. Under stress conditions transcriptional remodeling of the niche has been linked to an adipocytic skewing of perivascular cells and vascular *Dll4* absence has led to a premature skewing of HSPCs toward a myeloid transcriptional program (Tikhonova et al., 2019).

Together with perivascular MSCs, ECs control HSPCs maintenance and leukocyte trafficking by forming a network of blood vessel types with distinct permeability properties. Heterogeneity amongst the contribution of the EC subpopulations to the stem cell niches has been revealed. Deletion of *Scf* in AECs, but not in SECs, has led to a reduction of functional HSCs (Xu et al., 2018). The highly permeable SECs promote HSPCs activation and constitute the site for leukocyte trafficking to and from the BM. The high permeability, associated with high reactive oxygen species (ROS) levels, increases HSPCs migration and differentiation, while compromising their long-term repopulation and survival. The less permeable arterial blood vessels maintain HSPCs in low ROS levels (Itkin et al., 2016). The establishment of unique perivascular micro-niches has been moderated by divergent localization to sinusoidal and arteriolar surfaces of CAR cell subsets (Adipo-CAR and Osteo-CAR) that mainly function as cytokine-producing cells (Baccin et al., 2020). Furthermore, live imaging of LT-HSCs in the mouse native niche defined a subset of highly quiescent LT-HSCs, residing close to both sinusoidal blood vessels and the endosteal surface. MPPs have been mainly linked to transition zone vessels. Steady-state LT-HSCs showed limited motility in contrast with activated LT-HSCs exhibiting high motility or clonal expansion in spatially restricted domains. These domains include BM cavities with remodeling features, where HSCs expansion takes place, and cavities with low bone-resorbing activity, lacking HSCs expansion, where the microenvironment might differ (Christodoulou et al., 2020). In addition to the significance of the intrinsic BM signals, extrinsic factors are also critical for HSC maintenance, as shown for thrombopoietin (TPO) expressed by hepatocytes (Decker et al., 2018).

Changes in BM niche might directly affect myeloid vs. lymphoid output. The niche changes substantially during aging (Lee G.Y. et al., 2019) and plays a major regulatory role in malignancies, where either alterations in BM can promote leukemic transformation or create a favorable microenvironment for malignant proliferation, though BM remodeling by LSCs. For example, LSCs can upregulate CXCR4 expression (Pinho and Frenette, 2019; Mendez-Ferrer et al., 2020). Different leukemia types can be linked with induction of specific niche remodeling alterations. Remodeling of BM stromal cell subpopulations in AML has been confirmed by single-cell RNA-seq. These findings support a model where the malignant cells alter differentiation of the surrounding stromal cells and decrease the expression of signaling molecules regulating normal hematopoiesis. The malignant clone competes with the normal hematopoietic cells, creating a less supportive environment (Baryawno et al., 2019). Further characterization of the niche heterogeneity will provide additional insights on the control of HSC quiescence vs. proliferation in young, aged and malignant conditions.

## JAK-STAT Pathway in Normal Hematopoiesis and Hematologic Malignancies

### JAK-STAT in Early Hematopoiesis

The JAK-STAT is amongst the most conserved signaling pathways allowing communication between the extracellular environment and the nucleus. It can be activated by a plethora of cytokines, growth factors and hormones and regulates proliferation, differentiation, migration and cell survival depending on the cellular context and the environmental stimuli (Harrison, 2012). JAK-STAT is important in developmental and homeostatic processes including, stem cell maintenance, hematopoiesis and immune cell development. The JAK family of kinases includes JAK1, -2, -3, and TYK2 (Firmbach-Kraft et al., 1990; Krolewski et al., 1990; Wilks et al., 1991; Takahashi and Shirasawa, 1994). STAT protein family in mammals includes STAT1, -2, -3, -4, -5a, -5b, -6, which contain a conserved structure (Ihle, 1996, 2001; Darnell, 1997). Ligand binding to the receptor allows JAK phosphorylation and activation that leads to phosphorylation of the receptor, acting as a docking site for the STATs that are subsequently phosphorylated by JAKs. This leads to the formation of STAT homodimers or/and heterodimers that translocate to the nucleus and bind to DNA to regulate transcription.

JAK2 activation, by several hematopoietic and other cytokines, leads to phosphorylation of STATs (Bousoik and Montazeri Aliabadi, 2018), including STAT5 that regulates HSCs proliferation, survival and self-renewal (Wang and Bunting, 2013). JAK1 and JAK2 are essential for HSC homeostasis. Conditional *Jak1* deletion in HSCs *in vivo* reduced their self-renewal capacity and modified lympho-myeloid differentiation (Kleppe et al., 2017), whereas *Jak2* knock-out is embryonic lethal due to ineffective erythropoiesis (Neubauer et al., 1998; Parganas et al., 1998). Conditional *Jak2* knock-out leads to BM failure, increased apoptosis and loss of quiescence in HSC-enriched Lin^–^Sca-1^+^c-Kit^+^ cells, confirming its critical role for HSCs maintenance and function (Akada et al., 2014). JAK3 has been found essential for innate lymphoid cell development (Robinette et al., 2018) and TYK2 for B-lymphoid tumors regulation (Stoiber et al., 2004).

STAT1 plays an important role in megakaryopoiesis (Huang et al., 2007). Activated STAT3 has promoted HSC self-renewal, under stimulated but not homeostatic states, rendering STAT3 significant for hematopoietic regeneration (Chung et al., 2006). STAT3 phosphorylation is required for the IFN-β induced apoptosis in primary pro-B cells (Gamero et al., 2006). Selective activation of STAT5 confirmed its role in the self-renewal of normal and leukemic stem cells (Kato et al., 2005). STAT5, through survival effects on HSCs, supports the hematopoietic reserve and promotes multilineage hematolymphoid development. STAT5A/5B-deficient mice show an impaired hematopoietic potential in diverse blood lineages (Snow et al., 2002). Induction of high STAT5A activity levels impaired myelopoiesis and induced erythropoiesis in CD34^+^ cells, while intermediate levels resulted in maximum proliferation (Wierenga et al., 2008). Distinct cytokine responses in STAT5 phosphorylation at the single-cell level of leukemic and normal progenitors exist (Han et al., 2009). STAT5A and STAT5B possess distinct cell-growth-promoting properties that differentially affect the biological activity of HSPCs. STAT5A phosphorylation at Ser779/780 (mouse/human) controls proliferation and transformation/expansion of HSPCs with higher potency than STAT5B (Ghanem et al., 2017). Other STATs are also involved in normal and leukemic hematopoiesis. For instance, CD38 expression in the BM microenvironment of multiple myeloma cells is regulated by both STAT1 and STAT3 (Ogiya et al., 2020).

### JAK-STAT in Hematologic Malignancies

Since the 1990s numerous studies have confirmed the association between activating mutations in JAK-STAT and hematologic disorders (Leonard and O’Shea, 1998; Levine et al., 2007; Jatiani et al., 2010). Such mutations leading to constitutive activation of JAK-STAT can occur upstream or within the molecular components of the pathway. These include mutations of the transmembrane receptors, the JAKs or other upstream oncogenes, the STATs and the autocrine/paracrine cytokine production, which collectively leads to STAT activation (O’Shea et al., 2015). Deregulated JAK/STAT signaling due to *JAK1* and *JAK3* somatic mutations has been observed in Cutaneous T-Cell Lymphoma (CTCL) (Perez et al., 2015). Translocations of the *JAK2* gene or the *JAK2*V617F mutation are underlying causes of hematological malignancies (Baxter et al., 2005; James et al., 2005; Jones et al., 2005; Levine et al., 2005). *JAK2*V617F is an activating point mutation resulting in increased JAK2 activity, leads to STAT5 activation (Levine et al., 2005), and has been described in the majority of patients with myeloproliferative neoplasms (MPNs). It has been detected in almost all patients with polycythemia vera (PV) and about 50% of the patients with essential thrombocytosis and primary myelofibrosis (Baxter et al., 2005; Passamonti and Maffioli, 2016). In PV patients the mutation occurs in HSCs and predisposes toward erythroid differentiation (Jamieson et al., 2006). Mouse models have contributed to the understanding of the mechanisms by which JAK-STAT or related mutations promote hematopoietic malignancies (Dunbar et al., 2017). Expression of *Jak2*V617F in BM progenitors resulted in a PV-like syndrome with myelofibrosis in a mouse BM transplant model (Wernig et al., 2006). Use of Pf4-Cre transgenic mice to drive *Jak2*V617F expression in megakaryocyte lineage-committed cells, augmented erythropoiesis and stimulated fibrosis, resulting in a myeloproliferative state. These findings confirmed that JAK/STAT activation in megakaryocytes induced myeloproliferation and is essential for MPN maintenance *in vivo* (Woods et al., 2019). Xenograft mouse models have also contributed to the understanding of JAK/STAT mechanisms in leukemia. For example, the importance of JAK/STAT in early T-cell precursor (ETP) acute lymphoblastic leukemia (ALL) has been confirmed when the JAK1/2 inhibitor ruxolitinib has been used in murine xenograft models leading to abrogation of the STAT5 activation in response to IL-7 (Maude et al., 2015).

STAT1, STAT3, and STAT5 have been found, since the 1990s, constitutively activated in cells from acute leukemias (Gouilleux-Gruart et al., 1996, 1997). STAT1 has been defined as a tumor promoter in leukemia development (Kovacic et al., 2006). STAT5 contributes to the development of malignancies influencing myeloid and lymphoid lineages. A constitutively activated STAT5A mutant, forming enhanced levels of stable tetramers has caused multilineage leukemias, with STAT5 tetramers to accumulate in excess to dimers in human leukemias (Moriggl et al., 2005). STAT5A Ser725 and 779 phosphorylation detected in human leukemic cell lines and primary patient samples has been found essential for hematopoietic cell transformation (Friedbichler et al., 2010). Additionally, the N-terminus of STAT5A/B is functionally important in B-lymphoid transformation (Hoelbl et al., 2006).

Myelodysplastic syndromes (MDS), a heterogeneous group of clonal disorders of HSCs with a risk of progression to AML (Sperling et al., 2017; Cazzola, 2020; Garcia-Manero et al., 2020), have complex molecular pathogenesis due to the high genomic heterogeneity (Awada et al., 2020). The development of AML is considered a multi-cause and -step process (Gruszka et al., 2017). Translocations and inversions including fusion oncogenes, that use the JAK-STAT pathway, have been involved. Initial activating mutations in receptor tyrosine kinases (e.g., FLT3) promote proliferation of hematopoietic progenitors and subsequently additional mutations targeting transcription factors and impairing differentiation and apoptosis are required to result in leukemic cells (Gilliland, 2002; Gruszka et al., 2017). *FLT3* is among the most commonly mutated genes in AML (Kiyoi et al., 2002; Ley et al., 2013). AML-specific *Flt3* mutations have induced STAT target genes (Mizuki et al., 2003) and *FLT3*-D835 mutation has led to constitutive activation of STAT5 (Taketani et al., 2004). Levels of CDC25A, a phosphatase important for proliferation and differentiation in AML expressing the *FLT3*-ITD mutation, are controlled by a complex STAT5/miR-16 transcription and translation pathway, confirming that *FLT3*-ITD/STAT5/miR-16/CDC25A interplay is important for AML cell proliferation and differentiation (Sueur et al., 2020). Furthermore, induced inflammatory response in the human AML niche leads to increased activity of the JAK/STAT pathway in AML blasts and BM stromal cells promoting leukemic proliferation (Habbel et al., 2020). An imatinib-upregulated lncRNA family has been identified as a negative regulator of Bcr-Abl-induced tumorigenesis, through suppression of the STAT5-CD71 pathway (Wang et al., 2019). STAT5B has been defined as more important than STAT5A in BCR/ABL-induced leukemia, explaining the high frequency of STAT5B mutations in hematopoietic malignancies (Kollmann et al., 2019). High activity levels of STAT5A and STAT5B variants in the hematopoietic system of transgenic mice can lead to a lethal condition resembling human peripheral T-cell lymphoma (PTCL) and elevated expression of STAT5A/B has been detected in human PTCL samples. Both factors have been confirmed as oncogenes in PTCL, with STAT5B to be more transforming (Maurer et al., 2020). Mutations in *STAT3* (Koskela et al., 2012) and *STAT5B* genes have been detected in large granular lymphocytic (LGL) leukemia patients, with the *STAT5B*N642H mutation to be connected with unfavorable disease progression (Rajala et al., 2013). The same mutation has been commonly found in pediatric T-cell acute lymphoblastic leukemia (T-ALL) and is linked to a higher risk of relapse (Bandapalli et al., 2014). Recently, a key contributor to B-cell lymphopoiesis, Early B cell factor 1 (EBF1), has been shown to possess an inhibitory role in chronic lymphocytic leukemia (CLL) through inactivation of the STAT5 pathway (Wang et al., 2021).

These findings confirm the functional involvement of mutated/activated STATs, miRNAs, and lncRNAs in hematologic malignancies. Numerous studies have identified target genes regulated by STATs in normal and leukemic settings (Theodorou et al., 2013; Nanou et al., 2017). Developments in next-generation sequencing at the multi- and single-cell level have contributed to the acceleration of such identifications. Genes, lncRNAs, miRNAs targeted by STAT factors are useful in stratification strategies, management of leukemia and provision of novel therapeutic targets.

## Therapeutic Implications: HSCs Transplantation and JAK-STAT Inhibitors

Hematopoietic stem cells are extensively utilized in advanced regenerative medicine therapies (Dessie et al., 2020). Cell damage in hematological malignancies can be restored by HSCs transplantation (HSCT). Advancements in transplant immunology led to decreased transplant-associated mortality and more effective HSCT. Efforts regarding allogeneic HSCT mainly focus on conditioning therapies, donor selection, and stem cell sources. The combination of graft-vs.-leukemia effector cells contained in the stem cell graft with advances on the human leukocyte antigen system allowed enhanced antitumor effect and improved donor selection (Juric et al., 2016). Alternative stem cell sources including granulocyte-colony stimulating factor-mobilized peripheral blood stem cells and cord blood cells have been also validated. Genetically modified T-cells expressing chimeric antigen receptors (CARs) specific for a selected tumor antigen, such as CD19 in B-cell malignancies, have been also introduced as more effective antileukemic cell-based approaches. Gene-editing tools including transcription activator-like effector nucleases (TALEN) and clustered regularly interspaced short palindromic repeats (CRISPR) (Li et al., 2020) resulted in eliminated alloreactivity and decreased immunogenicity. However, further optimizations are needed, and many challenges still exist.

The JAK-STAT pathway constitutes a promising target for the development of various indirect and direct inhibitors for malignancies (Springuel et al., 2015; Brachet-Botineau et al., 2020). Indirect inhibitors focus on approaches using upstream tyrosine kinases targeting, natural and synthetic molecules, and drug repositioning. The understanding of the *JAK2*V617F mutation mechanism and the elaboration of the pseudokinase domain structure has provided the opportunity for the development of JAK2 inhibitors for MPN treatment targeting only the mutated kinase, as JAK2 is necessary for normal hematopoiesis. The first selective JAK inhibitor (JAKinib) to be tested and later approved in humans has been Tofacinitib, which targets JAK1, JAK2 and JAK3 (Kontzias et al., 2012; O’Shea et al., 2015). Ruxolitinib, the first JAKinib approved by the United States Food and Drug Administration (FDA), is a potent inhibitor of JAK1 and JAK2, used for primary myelofibrosis (O’Shea et al., 2015) and its effects have been also studied in MDS, AML, ALL, chronic myelomonocytic leukemia (CMML) and chronic myeloid leukemia (CML) (Eghtedar et al., 2012; Pemmaraju et al., 2015). JAKinibs might also ameliorate treatment by monoclonal antibody therapies for myeloma patients. This represents a novel therapeutic option, as Ruxolitinib inhibition of the JAK-STAT3 pathway has been shown to increase CD38 expression and anti-CD38 monoclonal antibody-mediated cytotoxicity (Ogiya et al., 2020). Another class of indirect inhibitors includes the first- and next-generation FLT3 inhibitors for AML. First-generation inhibitors lack specificity. Next-generation inhibitors have higher specificity, potency, lower toxicities and are under clinical investigation for AML (Daver et al., 2019). Recently an inhibitor targeting Aurora A (AKI604), has been shown to block the leukemic proliferation induced by STAT5, thus suggesting that the use of Aurora kinase inhibitors (AKIs) might be promising to overcome STAT-induced leukemic proliferation in AML (Wang et al., 2020).

Several natural and synthetic compounds exerting anti-tumor functions through their action on STAT3 and/or STAT5 signaling have been developed. These low toxicity compounds can synergize with other pharmacological agents to reverse chemoresistance. For example, the inhibitor 17f has been shown to selectively inhibit STAT5 signaling in CML and AML cells (Brachet-Botineau et al., 2019). Resveratrol, a naturally occurring plant compound, inhibited STAT5 activation in CML cell lines, providing a potential CML treatment (Li et al., 2018).

For drug repositioning, cell-based assays for high-throughput screening have been employed to identify compounds specifically inhibiting STAT3/5 transcriptional activity. For instance, pyrimethamine, an antimalaria drug, previously identified as a STAT3 signaling inhibitor, provided a potential AML treatment (Takakura et al., 2011; Sharma et al., 2016).

Direct inhibitors of STAT3/5 include molecules obstructing tyrosine phosphorylation, dimerization, nuclear translocation and/or DNA binding. Inhibitors targeting STAT3/5 domains or mRNAs have been developed (Brachet-Botineau et al., 2020). Nucleic acid based inhibition strategies include antisense oligonucleotides (ASO), siRNA, dominant-negative constructs, G-quartet oligonucleotides and decoy oligonucleotides (Sen and Grandis, 2012). AZD9150, an ASO targeting STAT3 mRNA, has decreased viability in leukemic cell lines (Shastri et al., 2018) and is now in phase 1/2 clinical trials (Brachet-Botineau et al., 2020).

## Conclusion

Research on HSCs and the BM niche has shed light on normal and leukemic hematopoiesis, however, their molecular intricacies have not been fully delineated. The developments in the field of single-cell omics have enhanced the understanding of the cellular and molecular organization of the niche bringing us a step closer to a more detailed functional characterization to improve HSCT and to discover novel therapeutic strategies for leukemia. Applied induction of effector CAR immune cells from HSCs can produce large numbers of immune cells for clinical evaluation. Gene therapy using autologous HSCs overcame the major issue of donor compatibility and ongoing research will further optimize the therapeutic dosage control, the low cell targeting and the retention in malignancy sites, however, many challenges remain to fully treat leukemia and its relapse (Chu et al., 2020). Research findings on the interconnections between HSCs-niche and signaling pathways (i.e., JAK-STAT) will further contribute to new approaches in stem cell engineering, HSCT and combinations with pharmacological approaches to improve safety and efficacy.

**FIGURE 2:**
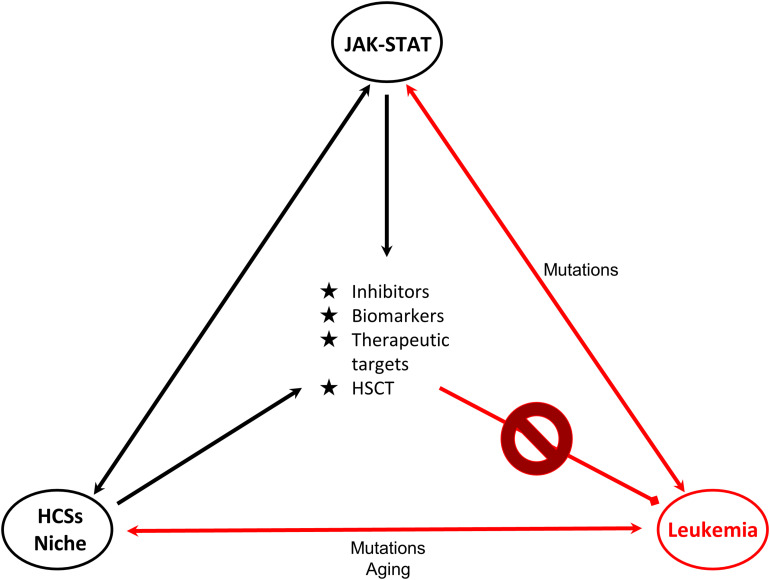
Connections of JAK-STAT, HSCs and leukemia. Schematic representation of the connections of HSCs-bone marrow niche, JAK-STAT pathway and leukemia is shown. JAK-STAT pathway regulates HSCs proliferation, survival and self-renewal, and components of the BM microenvironment. Mutations linked to JAK-STAT and/or HSCs-niche can lead to leukemic transformation. Research findings on these connections provide opportunities (in the middle of the triangle) for the management and therapy of leukemia. Double arrows represent bidirectional connections. For example mutations in JAK-STAT can cause leukemia, but also in leukemia cells deregulated JAK-STAT pathway is observed. HSC: Hematopoietic stem cell, HSCT: Hematopoietic stem cell transplantation.

The delineation of the role of the JAK/STAT pathway in hematologic malignancies rendered its components ideal candidates for the development of novel therapeutic strategies. STAT5, a significant signaling regulator in normal HSCs and LSCs constitutes an attractive candidate for innovative therapies. Combinations of JAKinibs with STAT inhibitors, monoclonal antibodies, growth factor support, hypomethylating agents, chemotherapy and allogeneic HSCT might be beneficial. Pyrimethamine, a direct inhibitor of activated STAT3, conjugated with histone deacetylase inhibitors, also known to inhibit STAT3 activation, has been used successfully in a breast cancer cell line for HDAC and STAT3 pathway inhibition (Wu et al., 2020). It cannot be excluded that conjugated inhibitors might also provide novel therapeutic solutions for hematologic malignancies. Targeting the communication between leukemia-initiating cells and their microenvironment together with the JAK-STAT pathway might be more effective and might overcome problems of inhibitor persistence and resistant subclones (Springuel et al., 2015). Furthermore, identification of genes, miRNAs, lncRNAs and other non-coding RNAs targeted by STATs will provide novel targets for therapies and useful biomarkers for monitoring of therapeutic strategies and patient stratification (Figure 2).

Although many new aspects and mechanisms of the hematologic malignancies have been revealed, further investigation is needed to define the role of JAK-STAT and the effects of BM niche in normal hematopoiesis, leukemia and aging. All the above will allow effective targeting of JAK-STAT and the development of personalized and accurate therapeutic management.

## Author Contributions

ESF and EK wrote and edited the manuscript. EK supervised manuscript preparation. Both authors contributed to the article and approved the submitted version.

## Conflict of Interest

The authors declare that the research was conducted in the absence of any commercial or financial relationships that could be construed as a potential conflict of interest.

## Abbreviations

HSCs: hematopoietic stem cells
JAK-STAT: Janus kinase-signal transducer and activator of transcription
BM: bone marrow
HSPCs: hematopoietic stem and progenitor cells
LT-HSCs: long-term self-renewing HSCs
ST-HSCs: short-term self-renewing HSCs
MPPs: multipotent progenitors
OPPs: oligopotent progenitors
AML: acute myeloid leukemia
LSC: leukemic stem cell
AGM: aorta-gonad-mesonephros
AECs: arterial endothelial cell
HECs: hemogenic endothelial cells
E: mouse embryonic day
EC: endothelial cell
MSCs: mesenchymal stem cells
SECs: sinusoidal ECs
OLCs: osteolineage cells
NES: nestin
SCF: stem cell factor
CXCL12: C-X -C motif chemokine 12
NG2: nerve/glial antigen 2
LEPR: leptin receptor
CAR: CXCL12-abundant reticular cells
Foxc1: forkhead box C1 protein
CXCL4: C-X -C motif chemokine 4
Dll4: Delta-like Notch ligand 4
ROS: reactive oxygen species
TPO: thrombopoietin
CXCR4: C-X -C motif chemokine receptor 4
TYK2: tyrosine kinase 2
IFN- β: interferon beta
Ser: serine
CTCL: cutaneous T-Cell lymphoma
MPNs: myeloproliferative neoplasms
PV: polycythemia vera
Pf4-Cre: Platelet factor 4-Cre recombinase
ETP: early T-cell Precursor
ALL: acute lymphoblastic leukemia
IL-7: interleukin 7
MDS: myelodysplastic syndromes
FLT3: fms-related receptor tyrosine kinase 3
FLT3-ITD: FLT3-internal tandem duplication
miR: microRNA
lncRNA: long non-coding RNA
PTCL: peripheral T-cell lymphoma
LGL: large granular lymphocytic
T-ALL: T-cell acute lymphoblastic leukemia
EBF1: early B cell factor 1
CLL: chronic lymphocytic leukemia
miRNA: microRNA
HSCT: HSCs transplantation
CARs: chimeric antigen receptors
TALEN: transcription activator-like effector nucleases
CRISPR: clustered regularly interspaced short palindromic repeats
JAKinib: JAK inhibitor
FDA: Food and Drug Administration
CMML: chronic myelomonocytic leukemia
CML: chronic myelogenous leukemia
AKIs: aurora kinase inhibitors
mRNA: messenger RNA
ASO: antisense oligonucleotides
siRNA: small interfering RNA
HDAC: histone deacetylase
NMSC: non-myelinating Schwann cells
LMPPs: lymphoid-primed multipotential progenitors
CLPs: common lymphoid progenitors
CMPs: common myeloid progenitors
MEPs: megakaryocyte/erythrocyte progenitors
GMPs: granulocyte/macrophage progenitors
NK-cells: natural-killer cells.
